# Perioperative complications in spinal trauma patients: does timing matter?

**DOI:** 10.1007/s00701-025-06442-6

**Published:** 2025-02-01

**Authors:** Charlotte Y. Adegeest, Cas J. Hilke, Godard C. W. de Ruiter, Mark P. Arts, Carmen L. Vleggeert-Lankamp, Raoul D. Martin, Wilco C. Peul, Paula Valerie ter Wengel

**Affiliations:** 1 Department of Neurosurgery, Leiden University Medical Center, Leiden, The Netherlands; 2 Department of Neurosurgery, Haaglanden Medical Center, The Hague, The Netherlands; 3 Department of Neurology, Spaarne Gasthuis, Haarlem, The Netherlands

**Keywords:** Spinal trauma, Surgical timing, Risk factors, Treatment, Spinal cord Injury

## Abstract

**Background:**

Early surgery in traumatic spinal fracture treatment may facilitate prompt mobilization, encountering affiliated complications. However, the safety and the benefits of early surgery are being questioned in spinal trauma patients. Therefore, the objective of this retrospective study is to investigate the effect of surgical timing on perioperative complications in these patients.

**Methods:**

Spinal trauma patients who underwent surgery between 2010 and 2020 in two Dutch Level-I trauma centers were included retrospectively and divided into an early (< 24 h), late (between 24 and 72 h) and delayed (> 72 h) surgical cohort. The primary outcome was the occurrence of peri-operative complications. Besides surgical timing, trauma and patient-specific factors were also analyzed as potential risk factors for the occurrence of complications.

**Results:**

A total of 394 patients were included, of whom 149 received early, 159 late and 86 delayed surgical treatment. The occurrence of perioperative complications was significantly associated with age, body mass index, comorbidities, ASA grade 3 and 4, spinal cord injury (SCI), AO Spine type C injury, additional chest injury, and surgical delay. A multivariable analysis showed that age, ASA category, AO Spine classification and SCI were significantly associated with perioperative complications. Moreover, a subsequent analysis in non-SCI patients demonstrated an association between perioperative complications and delayed surgery.

**Conclusions:**

In this study, delayed surgical treatment is potentially associated with more perioperative complications compared to early surgery in non-SCI patients. Other possible risk factors for the occurrence of perioperative complications may be older age, ASA 3 and 4, AO spine C injury and SCI.

## Introduction

Traumatic spinal fractures impose a substantial burden of disability among the adult population and occasionally necessitate surgical intervention [10, 11, 16]. Traditionally, these fractures were predominately seen in young people involved in traffic accidents [14]. However, a growing prevalence among elderly patients is observed in clinical practice [24]. In specific cases, spinal fractures can result in spinal cord injury (SCI) leading to neurological impairments. The primary surgical approach involves spinal fixation, occasionally accompanied by spinal cord decompression.

Current guidelines predominately focus on traumatic SCI patients receiving surgical intervention within 24 h after trauma. However, an ongoing debate revolves around surgical timing for spinal trauma [9]. Intervention in case of spinal injury aims prompt stabilization to prevent SCI and facilitate early mobilization. Existing literature presents inconsistencies, mostly due to the utilization of various time frames. While some studies suggest that early surgery is linked to decreased postoperative complications and shorter hospital stays [5, 12, 17, 23], contrasting findings are found that do not demonstrate this impact of surgical timing [13, 15, 25]. Consequently, a definitive guideline is lacking regarding the optimal timing of surgery for spinal fractures raising uncertainty about the urgency of surgical intervention [19].

In light of this gap, this retrospective study aims to examine differences in perioperative complications between patients who underwent early surgical treatment (< 24 h after trauma) and those who received late or delayed surgical treatment (> 24 h or > 72 h after trauma). Furthermore, this study seeks to identify the risk factors associated with occurrence of perioperative complications. By shedding light on these aspects, our study aims to provide valuable insights to enhance the existing knowledge on the management of traumatic spinal fractures.

## Methods

We conducted a retrospective cohort study on patients with traumatic spinal fractures who were surgically treated between January 2010 and April 2020 at Leiden University Medical Centre (LUMC), Leiden, and Haaglanden Medical Centre (HMC), the Hague, two Dutch level-1 trauma centers. Approval by the Medical Ethical Research Council was granted. Patients either arrived directly to the emergency room (ER) of the Level-1 trauma center or were transferred from a non-trauma center to the ER of the Level-1 trauma center. Patients were selected according to predefined inclusion and exclusion criteria described in Table 1. Patient data was collected including medical history and medication use, details of injury (including mechanism, timing, additional injury), imaging characteristics, surgical treatment and follow up data such as hospital length of stay (LOS), postoperative length of stay (pLOS), admission on the Intensive Care Unit (ICU), intubation and duration of immobilization.

**Table 1:** Inclusion and exclusion criteria

Inclusion criteria	Exclusion criteria
1) One or more spinal fractures	1) Pathological or other non-traumatic fractures
2) With and without SCI	2) Unknown date of trauma
3) Surgery as first treatment of choice	3) Missing data about surgical timing
4) Age > 15	4) Surgery performed after failed conservative treatment
	5) Neurotrauma (GCS < 12)

Abbreviations: *SCI *Spinal Cord Injury, *GCS *Glasgow Coma Scale

Surgical timing was defined as the time of presentation to the ER until the start of surgery. When patients were transferred from another center, the initial time of presentation at the referring ER was used. The time of presentation to the ER was taken as baseline to create a consistent manner for data collection as this is registered in the electronic patient file. Moreover, the mean time from accident to ER in patients with traumatic SCI in the Netherlands is approximately fifty minutes which is relatively short [18]. Obesity was defined as BMI ≥ 30 according to the WHO standard. The trauma mechanism was divided into high energy trauma (HET) and low energy trauma (LET). The comorbidities examined in this study included cardiovascular diseases, pulmonary disease (including chronic obstructive pulmonary disease), liver diseases, kidney diseases and malignancies. Due to the retrospective design of the study and the limited availability of information in many cases, it was not feasible to calculate comorbidity or frailty scores for each patient. To assess the patients overall health, the ASA score was utilized, as it was available for nearly all participants. Polytrauma was defined as the presence of additional injuries to the head, chest, abdomen or extremities.

### Statistical analysis

The primary outcome was the prevalence of perioperative complications in a time window from trauma up to thirty days after surgery. Perioperative complications were stratified into mild, severe and serious complications based on the Clavien Dindo classification [6].

Firstly, patients were stratified into an early cohort (< 24 h), a late cohort (between 24 and 72 h) and a delayed cohort (> 72 h). These timeframes were chosen in concordance with other studies on spinal fractures [1, 8]. Secondarily, the patients were stratified in groups based on the occurrence of perioperative complications. Thirdly, a multivariable regression analysis was conducted in the overall study population. In this analysis, interaction terms were introduced to assess whether the relationship between surgical timing and complication rate varied depending on the presence of polytrauma or SCI. This consideration arises from the possible influence of polytrauma and SCI on the decision for optimal surgical timing [21]. Eventually, regarding surgical timing, a differentiation was made between between early to late surgery (≤ 72 h) and delayed surgery(> 72 h). Finally, a subsequent multivariable regression analysis was performed on a subgroup excluding SCI patients to further explore the impact of surgical timing on complication rate in non-SCI patients. Because of the international guidelines advocating surgery within 24 h after trauma in SCI patients, a potential uneven distribution can exist among the surgical timing group in our study population between SCI and non-SCI patients using the 72 h cut-off point [8]. Moreover, SCI can potentially influence the development of perioperative complications due to prolonged immobility caused by neurological deficit [7, 25]. For these reasons, SCI-patients were excluded from the final, multivariable analysis.

Statistical analysis was performed using RStudio 4.2.1. Cases with missing data about a specific item were excluded from the analysis regarding that specific variable. The p-value for all statistics was set at *p* < 0.05 as significant. For continuous variables, normality, median and interquartile range were calculated. To examine the association between demographics and other patient variables with perioperative and intraoperative complications, linear and logistic regression analyses were used.

## Results

### Study population

A total of 394 patients met the inclusion criteria, of whom 241 were male and 153 were female (see Table 2). Early surgery was performed in 159 patients, whereas surgery between 24 and 72 h was performed in 149 patients, and delayed surgery was performed in 86 patients.

**Table 2:** General demographics

Number of patients (*n*)		394
Gender	*Male*	241
	*Female*	153
SCI	*Yes*	92
	*No*	302
Level	*Cervical*	241
	*Thoracic*	91
	*Lumbar*	62
Complications		151 (38.3%)
	*Mild (I-II)*	106 (26.9%)
	*Severe (III-IV)*	32 (8.1%)
	*Mortality (V)*	12 (3%)

Abbreviations: *SCI *spinal cord injury

### Risk factors

The overall complication rate was 38.3%. Complications observed included acute kidney injury, ileus, neurological deterioration, pneumonia, postoperative hemorrhage, postoperative wound infection, pressure ulcer, pulmonary embolism, sepsis and urinary tract infection. The frequency of each complication within distinct subgroups (≤ 72 h vs. > 72 h and polytrauma vs. non-polytrauma) is presented in Table 3. Analysis of heterogeneity in complication frequencies among these subgroups using Fischer’s exact tests yielded non-significant results of both surgical timing groups (*p* = 0.385) and polytrauma groups (*p* = 0.265). Risk factors for development of perioperative complications according to the univariate analysis were age, high body mass index (BMI), comorbidity, ASA 3 and 4, SCI, additional chest injury and AO type C injury (see Table 4). The majority of comorbidities were cardiovascular diseases or diabetes mellitus, however, the sample size of these patient groups were too small to conduct a subanalysis on specific comorbidities. Moreover, the time to surgery was significantly longer in the complication group compared to the non-complication group (62.4 h versus 47.9 h, *p* = 0.032). The reasons for delayed surgery were logistic issues (full operation schedules, availability of spine surgeons), patient-related delays and concomitant life-threatening injuries which needed more urgent treatment. Patient-related delays involved postponed patient presentation at the ER or a delay in undergoing imaging directly after trauma, due to various reasons. Also, the patients in the complication group were more frequently admitted to the ICU and had prolonged hospital LOS. Days of immobilization were significantly higher in the complication group in comparison to the non-complication group (5.5 d vs. 3.1 d, *p* < 0.001).

**Table 3:** Overview of complications in non-SCI population

	Surgical Timing^a^		Polytrauma^b^	
	*≤ 72 h*	*> 72 h*	*Yes*	*No*
Total patients (*n*)	212	75	142	160
Acute kidney injury (*n*, (%))	2 (0.9)	3 (4.0)	2 (1.4)	3 (1.9)
Ileus (*n*, (%))	5 (2.4)	1 (1.3)	2 (1.4)	5 (3.1)
Neurological deterioration (*n*, (%))	8 (3.8)	3 (4.0)	7 (4.9)	5 (3.1)
Pneumonia (*n*, (%))	11 (5.2)	11 (14.7)	11 (7.7)	13 (8.1)
Postoperative hemorrhage (*n*, (%))	1 (0.5)	0 (0.0)	0 (0.0)	1 (0.6)
Postoperative wound infection (*n*, (%))	12 (5.7)	2 (2.7)	9 (6.3)	7 (4.4)
Pressure ulcer (*n*, (%))	1 (0.5)	1 (1.3)	2 (1.4)	0 (0.0)
Pulmonary embolism (*n*, (%))	1 (0.5)	2 (2.7)	2 (1.4)	2 (1.3)
Sepsis (*n*, (%))	1 (0.5)	1 (1.3)	0 (0.0)	2 (1.3)
Urinary tract infection (*n*, (%))	9 (4.2)	5 (6.7)	7 (4.9)	9 (5.6)
Death (*n*, (%))	2 (0.9)	2 (2.7)	5 (3.5)	0 (0.0)

^a^Fischer’s exact test for differences in complication frequencies between the ≤ 72 h and > 72 h group was non-significant (*p* = 0.385)

^b^Fischer’s exact test for differences in complication frequencies between the polytrauma and non-polytrauma group was non-significant (*p* = 0.265)

**Table 4:** Demographics of patient groups divided based on occurrence of complication

	All patients			SCI patients excluded		
	No complication	Complication	*p*-value	No complication	Complication	*p*-value
*N*	252	163		206	96	
Age (median[IQR])	56.5 [36.0, 68.3]	67.0 [47.0, 77.0]	*< 0.001*	54.4	64.6	*< 0.001*
Time to surgery (h, mean (SD))	47.9 (44.8)	62.4 (88.2)	*0.032*	55.6 (45.8)	71.2 (86.5)	*0.047*
Male (%)	147 (58.3)	109 (66.9)	*0.100*	117 (56.8)	57 (59.4)	*0.766*
Smoker, yes (%)	40 (15.9)	26 (16.0)	*0.324*	30 (14.6)	14 (14.6)	*0.801*
BMI (%)			*0.001*			*0.002*
Normal	133 (52.8)	59 (36.2)		104 (50.5)	33 ( 34.4)	
Overweight	46 (18.3)	25 (15.3)		38 (18.4)	12 (12.5)	
Obese	20 (7.9)	18 (1.0)		18 (8.7)	10 (10.4)	
NA	53 (21.0)	61 (37.4)		46 (22.3)	41 (42.7)	
Comorbidity (%)	137 (54.4)	106 (65.0)	*0.040*	115 (55.8)	63 (65.6)	*0.137*
ASA (%)			*< 0.001*			*< 0.001*
ASA 1	72 (28.6)	14 (8.6)		59 (28.6)	10 (10.4)	
ASA 2	107 (42.5)	50 (30.7)		94 (45.6)	27 (28.1)	
ASA 3	36 (14.3)	49 (30.1)		31 (15.0)	34 (35.4)	
ASA 4	7 (2.8)	24 (14.7)		1 (0.5)	10 (10.4)	
Unknown	14 (5.6)	10 (6.1)		9 (4.4)	7 (7.3)	
NA	16 (6.3)	16 (9.8)		12 (5.8)	8 (8.3)	
SCI (%)			*< 0.001*			
No	206 (81.7)	96 (58.9)		-	-	
Yes	42 (16.7)	56 (34.4)		-	-	
NA	4 (1.6)	11 (6.7)		-	-	
Severity of SCI			*0.022*			
AIS A	7 (2.8)	14 (8.6)		-	-	
AIS B	9 (3.6)	8 (4.9)		-	-	
AIS C	4 (1.6)	7 (4.3)		-	-	
AIS D	17 (6.7)	13 (8.0)		-	-	
Polytrauma, yes (%)	118 (46.8)	88 (54.0)	*0.185*	96 (46.6)	46 (47.9)	*0.929*
Additional chest injury (%)	47 (18.7)	54 (33.1)	*0.001*	34 (16.5)	23 (24.0)	*0.167*
Additional abdominal injury (%)	11 (4.4)	15 (9.2)	*0.075*	6 (2.9)	9 (9.4)	*0.034*
Additional extremity injury (%)	57 (22.6)	43 (26.4)	*0.449*	47 (22.8)	23 (24.0)	*0.942*
Mechanism (%)			*0.925*			*0.756*
HET	151 (59.9)	96 (58.9)		121 (58.7)	52 (54.2)	
LET	97 (38.5)	65 (39.9)		83 (40.3)	43 (44.8)	
NA	4 (1.6)	2 (1.2)		2 (1.0)	1 (1.0)	
Level of injury (%)			*0.382*			*0.870*
Cervical	155 (61.5)	100 (61.3)		121 (58.7)	55 (57.3)	
Thoracic	54 (21.4)	42 (25.8)		46 (22.3)	24 (25.0)	
Lumbar	43 (17.1)	21 (12.9)		39 (18.9)	17 (17.7)	
AO Spine Classification (%)			*0.009*			*0.682*
Type A	81 (32.1)	40 (24.5)		67 (32.5)	26 (27.1)	
Type B	63 (25.0)	34 (20.9)		49 (23.8)	23 (24.0)	
Type C	48 (19.0)	55 (33.7)		33 (16.0)	20 (20.8)	
NA	60 (23.8)	34 (20.9)		57 (27.7)	27 (28.1)	
Admission on ICU, yes (%)	61 (24.2)	94 (57.7)	*< 0.001*	37 (18.0)	42 (43.8)	*< 0.001*
Number of days on ICU (mean (SD))	3.6 (4.2)	10.3 (13.1)	*< 0.001*	2.4 (1.8)	6.2 (7.5)	*0.003*
Intubation, yes (%)	18 (7.1)	47 (28.8)	*< 0.001*	7 (3.4)	15 (15.6)	*< 0.001*
Surgical time (h, %)			*0.020*			*0.030*
< 24 h	95 (37.7)	64 (39.3)		55 (26.7)	22 (22.9)	
24–72 h	103 (40.9)	46 (28.2)		99 (48.0)	36 (37.5)	
> 72 h	45 (17.9)	41 (25.2)		44 (21.4)	31 (32.3)	
NA	9 (3.6)	12 (7.4)		8 (3.9)	7 (7.3)	
Hospital length of stay (hLOS, median [IQR])	7.0 (4.0, 10.0)	14.0 (9.0, 30.0)	*< 0.001*	7.5 (5.3)	16.7 (12.6)	*< 0.001*
Number of postoperative days (median [IQR])	4.0 (2.0, 7.0)	13.0 (6.0, 27.5)	*< 0.001*	5.1 (5.0)	13.7 (12.2)	*< 0.001*
Number of days immobilized (mean (SD))	3.1 (1.7)	5.5 (5.0)	*< 0.001*	3.2 (1.7)	4.6 (3.4)	*< 0.001*

Abbreviations: *BMI *body mass index, *ASA *American Society of Anesthesiologists, *SCI *spinal cord injury, *ICU *Intensive Care Unit, *hLOS *hospital length of stay

Excluding the SCI patients, similar outcomes in the univariate analysis were observed, with the exception of no notable difference in AO Spine Type C injury, comorbidity, and additional chest injury (see Table 4). Nevertheless, ASA 3 and 4 and delayed surgery continued to emerge as a risk factor, and there appeared to be an increased risk of complications in case of additional abdominal injury.

### Surgical timing

Patients who underwent early surgery were slightly younger, but this difference was non-significant (59.0 y vs. 61.0 y and 62.5 y, *P* = 0.070) (see Table 5). Patients in the early surgery group more frequently suffered from SCI (46.5% vs. 9.4% and 4.7%, *p* < 0.001) and AO Spine Type C injury (34.6% vs. 21.5% and 9.3%, *p* < 0.001), while patients in the delayed surgery group exhibited more frequently additional chest (33.7% vs. 25.8% and 16.1%, *p* = 0.007), abdominal (14.0% vs. 3.1% and 4.7%, *p* = 0.002) or extremity injury (32.6% vs. 17.6% and 24.8%, *p* = 0.028) (see Table 5). Only four SCI patients received surgery surpassing 72 h, while 88 SCI patients received surgery within 72 h. The complication rates also differed significantly between each group, where the greatest complication rate was in the delayed surgery group (47.7%), followed by early surgery group (40.3%) and late surgery group (30.9%). Contrarily, the most severe complications (17.6% vs. 5.4% and 9.3%, *p* = 0.002) and the highest mortality rate (6.3% vs. 0% and 2.3%, *p* = 0.005) were mainly in the early surgery group. Two patients died of sepsis during hospital admission. Two patients died because of concomitant injury. In six patients, a consensus was reached with the patient and family to initiate palliative treatment primarily prompted by respiratory failure due to high cervical SCI. Finally, a significant difference was found between days of immobilization (3.0 vs. 3.5 vs. 6.1, *p* < 0.001), postoperative days (8.0 vs. 5.0 vs. 6.0, *p* < 0.001) and hospital LOS (9.0 vs. 7.0 vs. 12.0, *p* < 0.001) between the three groups.

**Table 5:** Demographics of patient groups divided based on surgical timing

	< 24 h	24–72 h	≥ 72 h	*p*-value	Test
*N*	159	149	86		
Age (median[IQR])	59.0 [36.0, 69.0]	61.0 [46.0, 74.0]	62.5 [44.3, 75.0]	*0.070*	Non-norm
Male (%)	109 (68.6%)	83 (55.7)	49 (57.0)	*0.046*	
Smoker, yes (%)	26 (16.4)	22 (14.8)	14 (16.3)	*0.997*	
BMI (%)				*0.539*	
Normal	77 (48.4)	71 (47.7)	33 (38.4)		
Overweight	28 (17.6)	27 (18.1)	15 (17.4)		
Obese	15 (9.4)	15 (10.1)	7 (8.1)		
NA	39 (24.5)	36 (24.2)	31 (36.0)		
Comorbidity (%)	88 (55.3)	96 (64.4)	51 (59.3)	*0.267*	
ASA (%)				*0.421*	
ASA 1	31 (21.4)	33 (22.1)	16 (18.6)		
ASA 2	60 (37.7)	62 (41.6)	31 (36.0)		
ASA 3	26 (16.4)	34 (22.8)	20 (20.3)		
ASA 4	15 (9.4)	6 (4.0)	8 (9.3)		
Unknown	10 (6.3)	9 (6.0)	5 (5.8)		
NA	14 (8.8)	5 (3.4)	6 (7.0)		
SCI (%)				*< 0.001*	
No	77 (48.4)	135 (90.6)	75 (87.2)		
Yes	74 (46.5)	14 (9.4)	4 (4.7)		
NA	8 (5.0)	0 (0.0)	7 (8.1)		
Severity of SCI				*< 0.001*	
AIS A	21 (13.2)	0 (0.0)	0 (0.0)		
AIS B	14 (8.8)	1 (0.7)	1 (1.2)		
AIS C	8 (5.0)	2 (1.3)	1 (1.2)		
AIS D	20 (12.6)	9 (6.0)	1 (1.2)		
Polytrauma, yes (%)	75 (47.2)	71 (47.7)	48 (55.8)	*0.385*	
Additional chest injury (%)	41 (25.8)	24 (16.1)	29 (33.7)	*0.007*	
Additional abdominal injury (%)	5 (3.1)	7 (4.7)	12 (14.0)	*0.002*	
Additional extremity injury (%)	28 (17.6)	37 (24.8)	28 (32.6)	*0.028*	
Mechanism (%)				*0.867*	
HET	94 (59.1)	90 (60.4)	50 (58.1)		
LET	62 (39.0)	58 (38.9)	34 (39.5)		
NA	3 (1.9)	1 (0.7)	2 (2.3)		
Level of injury (%)				*0.273*	
Cervical	99 (62.3)	94 (63.1)	48 (55.8)		
Thoracic	39 (24.5)	27 (18.1)	25 (29.1)		
Lumbar	21 (13.2)	28 (18.8)	13 (15.1)		
AO Spine Classification (%)				*< 0.001*	
Type A	37 (23.3)	47 (31.5)	28 (32.6)		
Type B	42 (26.4)	31 (20.8)	22 (25.6)		
Type C	55 (34.6)	32 (21.5)	8 (9.3)		
NA	25 (15.7)	39 (26.2)	28 (32.6)		
Admission on ICU (%)	73 (45.9)	40 (26.8)	34 (39.5)	*0.002*	
Number of days on ICU (mean (SD))	8.4 (12.6)	4.2 (6.6)	9.0 (8.2)	*0.075*	
Intubation (%)	34 (21.4)	12 (8.1)	16 (18.6)	*0.014*	
Complications (%)	64 (40.3)	46 (30.9)	41 (47.7)	*0.031*	
Severity of complications (%)				*0.002*	
No complication	95 (59.7)	103 (69.1)	45 (52.3)		
Mild (I-II)	36 (22.6)	37 (24.8)	33 (38.4)		
Severe (III-IV)	28 (17.6)	8 (5.4)	8 (9.3)		
Mortality (%)	10 (6.3)	0 (0.0)	2 (2.3)	*0.005*	
Hospital length of stay (hLOS, median [IQR])	9 [4.0, 18.5]	7 [5.0, 11.0]	12 [7.3, 17.0)	*< 0.001*	
Number of postoperative days (median [IQR])	8 [4.0, 18.0]	5 [3.0, 9.0]	6 [3.0, 12.5]	*< 0.001*	
Number of days immobilized (mean (SD))	3.0 (3.4)	3.5 (2.4)	6.1 (3.6)	*< 0.001*	

Abbreviations: *BMI *body mass index, *ASA *American Society of Anesthesiologists, *SCI *spinal cord injury, *ICU *Intensive Care Unit, *hLOS* hospital length of stay

### Multivariable regression analysis

Data of 252 patients was available for the multivariable analysis. In this analysis, conducted in the total study population, factors such as age, ASA 3 and 4, AO type C injury and SCI were significantly associated with the occurrence of perioperative complications (Table 6). Conversely, delayed surgery (> 72 h) and polytrauma as well as their interactions terms with surgical timing did not demonstrate significance. However, the multivariable analysis in the non-SCI subgroup revealed that, in addition to age, ASA 3 and 4 and AO Spine type C injury, delayed surgery (> 72 h) exhibited a significant association with the occurrence of perioperative complications (Table 6). The interaction term between surgical timing and polytrauma remained non-significant.

**Table 6:** Multivariable analysis of risk factors for occurrence of perioperative complications in the total study population and in non-SCI patients

	Total population			Non-SCI patients		
Variable	n	OR (95% CI)	*p*-value	*n*	OR (95% CI)	*p*-value
Age						
< 65 y	168	Ref		122	Ref	
≥ 65 y	84	2.53 (1.31, 4.93)	*0.006*	64	3.25 (1.47, 7.31)	*0.004*
ASA category						
ASA I & II	182	Ref		138	Ref	
ASA III & IV	70	3.25 (1.67, 6.40)	*< 0.001*	48	3.95 (1.76, 9.03)	*< 0.001*
AO Spine Classification						
AB	178	Ref		143	Ref	
C	74	2.13 (1.11, 4.14)	*0.024*	43	2.34 (0.98, 5.67)	*0.055*
Surgical Timing^a, b^						
< 72 h	207	Ref		144	Ref	
≥ 72 h	45	2.14 (0.15, 54.38)	*0.578*	42	4.16 (1.43, 12.58)	*0.010*
Polytrauma^a, b^						
Yes	125	Ref		92	Ref	
No	127	0.75 (0.38, 1.46)	*0.394*	94	0.92 (0.38, 2.21)	*0.842*
Spinal Cord Injury^a^						
Yes	66	Ref				
No	186	0.30 (0.15, 0.60)	< 0.001			

Abbreviations: *ASA *American Society of Anesthesiologists

^a^Interaction terms Surgical Timing-Polytrauma and Surgical Timing-Spinal Cord Injury are non-significant

^b^Interaction term Surgical Timing-Polytrauma is non-significant

## Discussion

This study shows that age, ASA category 3 and 4, AO Spine type C injury and SCI are associated with a higher risk of perioperative complications. Furthermore, our results indicate that delayed surgery can possibly cause an increased risk of perioperative complications, especially in non-SCI patients.

### Risk factors

In our study, we identified age, high BMI, ASA category 3 or 4, SCI, additional chest injury and AO Spine C injury as risk factors. These findings are consistent with results from similar studies. Sreeharsa et al. reported an increased number of perioperative complications in older patients (> sixty years) and in those with complete SCI or additional chest injury [25]. Similarly, a recent study by Cabrera et al. on complications in surgically treated thoracolumbar AO Spine B and C injury patients further underscored the predictive role of surgical timing beyond 72 h, along with comorbidity, complete SCI and AO Spine Type C injury [3]. However, a study by Dimar et al., focusing on thoracolumbar spine fractures, showed no influence of surgical timing on complication rate, but highlighted the impact of SCI and comorbidities on complication occurrence [7].

Our study reaffirms the significance of age as a risk factor for the occurrence of perioperative complications, which may be coherent with comorbidity. Given the global trend towards an aging population, it is of great importance to understand the safest and most effective treatment strategies for elderly patients with spinal fractures [4]. By using the ASA score as an indicator of overall patient health, along with age, our study suggests that elderly patients (> 65 years) with higher comorbidity (ASA 3 and 4) are at greater risk for complications compared to younger, healthier patients. However, further research is needed to determine whether specific comorbidities contribute to an increased complication rate.

Other potential risk factors found in our analysis were additional chest injury and SCI. Both features are predisposing elements for perioperative complications due to their correlation with poor respiratory function. This respiratory disfunction often necessitates extended periods of ventilatory support, which can subsequently result in prolonged immobility and thereby contributes to the onset of (pulmonary) infections and further heightened morbidity [25, 28].

### Surgical timing

The outcomes of the univariate and non-SCI multivariable analyses in our study emphasized that surgical delay surpassing 72 h was significantly linked to a higher risk of perioperative complications compared to earlier surgery. Within the total study population, the multivariable analysis did not reveal a significant association between surgical timing and complication rate. This lack of significance may be attributed to the imbalanced distribution of SCI patients across the surgical timing categories (*n* = 88 ≤ 72 h vs. *n* = 4 > 72 h), which is supported by the wide confidence interval (95%CI 0.15–54.38). This disproportionate division of SCI patients among the surgical timing groups may also account for the non-significance of the interaction term.

Furthermore, patients subjected to delayed surgery experienced prolonged LOS and immobilization periods. It is worth noting that extended LOS and immobilization duration might also be attributed to an increased number of pre-operative days in the delayed surgery group, including a prolonged period of immobilization.

While several studies have reported a reduction in LOS for the early surgery group compared to delayed surgeries [13, 15, 25], a significant connection between surgical timing and perioperative complications has not been consistently established. However, a study by Pakzad et al. found a significant association between early surgery (< 24 h) and reduced perioperative complications in patients with spinal fractures without neurological deficit [20]. Boakye et al. further underscored the significance of surgical timing as the primary predictor for perioperative complications, followed by age, comorbidity and severity of injury [2]. It is worth noticing that while that study focused solely on thoracic or thoracolumbar injuries, our study encompassed spinal trauma across all levels. Finally, a study by Sousa et al. on the safety of early surgery (< 72 h) for spinal fractured patients admitted to the ICU, revealed a shortened ICU LOS and intubation time in case of early surgery, along with comparable mortality rates between the different groups [26]. Considering these findings, there seems to be a beneficial effect of early surgery in mitigating perioperative complications in patients with spinal fractures without SCI.

It is, however, important to acknowledge that surgical timing is often influenced by multiple factors. Firstly, one of the most important factors determining surgical timing is SCI due to the international guideline advocating surgery within 24 h after trauma [8]. This recommendation is evident in our study population, where 88 SCI patients received surgery within 72 h and only four SCI patients underwent delayed surgery (> 72 h). Secondly, the decision-making process for elderly patients can be intricate, leading to delays in surgical intervention. Factors often associated with advanced age such as comorbidity and anticoagulant usage, can pose challenges to pursuing early surgery. However, it is important to note that age, comorbidity and ASA scores did not significantly differ between the surgical timing groups (Table 5), indicating that these factors did not impact the surgical timing. Thirdly, the “treat-first-what-kills-first principle” applies to polytrauma patients who may necessitate urgent treatment for concurrent injuries, which can potentially delay spinal surgery. Also, hemodynamic instability due to additional chest or abdominal injuries can further complicate the feasibility of early surgical intervention, or can on itself increase the risk of complication development. Finally, it should be recognized that trauma itself triggers a heightened stress response, potentially leading to an inflammatory reaction of the body [26, 27, 29]. This reactive inflammation could be further exacerbated by surgical intervention resulting in adverse outcomes and increased mortality [27, 29, 30]. This multifactoriality of surgical timing contributes to the intricacy of risk assessment in this patient population, as each individual’s condition can be influenced by a range of variables, making it challenging to predict perioperative complications with certainty.

In our study population, it is notable that age and comorbidity did not significantly differ among the three surgical timing groups. However, patients in the delayed surgery group demonstrated a higher incidence of additional chest, abdomen or extremity injuries, potentially contributing to the increased complications observed in this group. For instance, additional chest injuries are associated with heightened risk of pulmonary complications [22]. While the presence of these factors hints at a possible indirect relationship between surgical timing and complications, more conclusive evidence is required. Nevertheless, an even distribution of the different complications within both the surgical timing groups (≤ 72 h vs. > 72 h) and the polytrauma groups (polytrauma vs. non-polytrauma) was demonstrated (see Table 3), which may contradict this argument.

### Limitations

Acknowledging the retrospective nature of our study, limitations arising from data collection relying on medical file comprehensiveness are inherent. Our results should therefore be interpreted with caution. In certain cases, the reasons behind surgical delays were unclear, potentially impacting the outcomes. Although the surgical timing was determined by the time of arrival at the ER, rather than the actual time of the accident, to the start of surgery, it is noteworthy that the duration from accident to ER arrival is relatively short in The Netherlands and registered with greater accuracy than the precise time of the accident itself [18]. Nevertheless, there exists variability in the interval between the accident and the presentation at the ER among different patients, leading to certain patients not being categorized correctly within their respective patient group which could influence the outcomes. However, this variability could not be reliably assessed using the information available in the patients records.

Furthermore, a mean age of sixty years in our study population is higher than comparative studies, which may influence outcomes. However, an aging population aligns with contemporary trends. Besides, age and comorbidity rates did not significantly differ between the three surgical timing groups.

Heterogeneity among the surgical timing groups may exist within this population, as it comprises individuals with SCI or additional injuries resulting from high energetic trauma, contrasted with patients solely presenting with isolated spinal fractures. mBoth polytrauma and SCI are potential risk factors for development of perioperative complications [7, 25], although the distribution of polytrauma patients among the various study groups did not significantly differ. Furthermore, no association was observed between polytrauma and complications in the multivariable analysis. The impact of SCI on complication development, however, is more evident in our analysis. Consequently, SCI patients were excluded from the second multivariable analysis.

Moreover, the rate of ICU admissions did differ between the patient groups, whereas the early surgery groups had increased ICU admissions and intubation events compared to the other groups. This could be declared by the fact that the majority of SCI patients received early surgery, whereas SCI patients are often admitted to the ICU for monitoring of hemodynamics and ventilation support. No differences between the groups regarding trauma mechanism or additional injury were found that could further explain the increased ICU admissions and intubation in the early surgery group. Ultimately, the intricate array of factors contributing to perioperative complications adds complexity to the attempt of distinguishing between complications resulting from surgical delays and those originating from alternate factors, such as intubation or concomitant injury.

Nevertheless, our findings may suggest that surgical intervention within 72 h post-trauma emerges a safer option, resulting in the lowest complication rate. However, comprehensive research is necessary to validate and refine these conclusions. Importantly, adaptation of the multidisciplinary hospital infrastructure of acute trauma care in the emergency room to the operation theatre is crucial to ensure surgical procedures can be executed within the 72-hours timeframe. Prospective studies focusing on perioperative complications in spinal injury patients, with detailed report on surgical delays, would offer deeper insights into the barriers influencing surgical timing within the 72-hous window. While our study emphasizes the benefits of early surgery, further prospective research is essential to validate and expand upon these findings.

## Conclusions

This study indicates that surgical timing possibly influences the occurrence of perioperative complications in (non-SCI) patients suffering traumatic spinal fractures. Other potential risk factors for the development of perioperative complications in spinal injury patients are age, comorbidity, AO Spine C injury and SCI. Nevertheless, the multifactorial nature of surgical timing necessitates consideration of factors such as age, comorbidity and concurrent injuries, which may influence surgical decision-making as well as the risk for perioperative complications. Prospective research is imperative to substantiate these findings and create optimal treatment guidelines.

## Data Availability

No datasets were generated or analysed during the current study.
